# The Influence of Parenting Style and Breastfeeding Attitudes on Child Behavior

**DOI:** 10.1192/j.eurpsy.2025.2022

**Published:** 2025-08-26

**Authors:** A. Kondyli, A. Zartaloudi, I. Koutelekos, D. Briana

**Affiliations:** 1 University of West Attica; 2 MSc in General Pediatrics and Pediatric Subspecialties - Clinical Practice and Research; 3 University of Athens, Athens, Greece

## Abstract

**Introduction:**

Parenting style plays a crucial role in shaping a child’s development and behavior. Supportive parenting has been associated with positive child outcomes, while authoritarian or permissive styles may lead to less desirable behaviors. Breastfeeding, known for its numerous benefits to both mother and child, may also interact with parenting approaches to influence a child’s emotional and social development.

**Objectives:**

To explore (a) the parenting style of the participants, (b) their attitudes towards breastfeeding, and (c) the behavioral characteristics of their children.

**Methods:**

A cross-sectional study was conducted using self-administered questionnaires completed by 862 parents—both mothers and fathers—who had received support from a private maternity and breastfeeding support center in Athens.

**Results:**

Mothers who adopted a more supportive parenting style had a more positive perception of their children’s behavior compared to more authoritarian, strict, or permissive mothers. Specifically, these supportive mothers viewed their children as less anxious (p = 0.015), more willing to share (p = 0.001), less irritable (p = 0.006), more affectionate (p = 0.009), more expressive of their emotions (p < 0.001), generally obedient (p < 0.001), better at maintaining attention (p < 0.001), less nervous in new situations (p = 0.019), and less easily frightened (p = 0.028). Fathers with a more supportive parenting style also perceived their children as sharing more readily (p < 0.001). Additionally, a longer duration of breastfeeding was associated with children who expressed their emotions more easily (p = 0.042) but were less obedient (p = 0.021). Finally, a more positive overall evaluation of the breastfeeding experience correlated with less agreement that the child is hyperactive (p = 0.020), irritable (p = 0.004), unreceptive to affection (p = 0.034), easily distracted (p = 0.004), and easily scared (p = 0.002).

**Conclusions:**

These findings highlight the importance of supportive parenting and positive breastfeeding attitudes in promoting favorable behavioral outcomes in children. Health professionals can use this information to encourage parenting practices that foster healthy child development.

**Disclosure of Interest:**

None Declared

